# Human cortical neurons rapidly generated by embryonic stem cell programming integrate into the stroke-injured rat cortex

**DOI:** 10.1093/stmcls/sxaf049

**Published:** 2025-07-12

**Authors:** Raquel Martinez-Curiel, Mazin Hajy, Oleg Tsupykov, Linda Jansson, Natalia Avaliani, Juliane Tampé, Emanuela Monni, Galyna Skibo, Olle Lindvall, Sara Palma-Tortosa, Zaal Kokaia

**Affiliations:** Laboratory of Stem Cells and Restorative Neurology, Lund Stem Cell Center, Lund University, Lund 22184, Sweden; Laboratory of Stem Cells and Restorative Neurology, Lund Stem Cell Center, Lund University, Lund 22184, Sweden; Department of Cytology, Bogomoletz Institute of Physiology, Kyiv 01024, Ukraine; Clinical and Regenerative Medicine, Institute of Genetic and Regenerative Medicine, Strazhesko National Scientific Center of Cardiology, Kyiv 01024, Ukraine; Laboratory of Stem Cells and Restorative Neurology, Lund Stem Cell Center, Lund University, Lund 22184, Sweden; Laboratory of Stem Cells and Restorative Neurology, Lund Stem Cell Center, Lund University, Lund 22184, Sweden; Laboratory of Stem Cells and Restorative Neurology, Lund Stem Cell Center, Lund University, Lund 22184, Sweden; Laboratory of Stem Cells and Restorative Neurology, Lund Stem Cell Center, Lund University, Lund 22184, Sweden; Department of Cytology, Bogomoletz Institute of Physiology, Kyiv 01024, Ukraine; Clinical and Regenerative Medicine, Institute of Genetic and Regenerative Medicine, Strazhesko National Scientific Center of Cardiology, Kyiv 01024, Ukraine; Laboratory of Stem Cells and Restorative Neurology, Lund Stem Cell Center, Lund University, Lund 22184, Sweden; Laboratory of Stem Cells and Restorative Neurology, Lund Stem Cell Center, Lund University, Lund 22184, Sweden; Laboratory of Stem Cells and Restorative Neurology, Lund Stem Cell Center, Lund University, Lund 22184, Sweden

**Keywords:** ischemic stroke, cell transplantation, human embryonic stem cells, induced neurons, restoration

## Abstract

Stem cell sources capable of producing appropriate cells for replacement will be necessary for functional repair of the injured brain. Here, we have determined whether transcription factor programming of human embryonic stem (hES) cells can be used to generate layer-specific cortical neurons capable of integrating into the stroke-injured rat cortex. Human embryonic stem cells were programmed via overexpression of neurogenin 2 (NGN2). After 7 days, hES-induced neurons (hES-iNs) were characterized *in vitro* using immunocytochemistry, RT-qPCR, and whole-cell patch-clamp. Cortical ischemic stroke was induced in rats via distal middle cerebral artery occlusion. Forty-eight hours later, hES-iNs were transplanted into the somatosensory cortex adjacent to the ischemic lesion. Three months thereafter, brains were analyzed for expression of neuronal markers, axonal myelination, and synapse formation using immunohistochemistry and immunoelectron microscopy (iEM). Overexpression of NGN2 in hES cells for 7 days generated excitatory neurons, expressing cortical markers at different stages of maturation. After transplantation, the hES-iNs expressed markers of both immature and mature neurons and of upper and deep cortical layers. The hES-iNs sent widespread projections to both hemispheres, and iEM revealed that they were myelinated by host oligodendrocytes and had formed efferent synaptic connections with host cortical neurons. The hES cells programmed via NGN2 overexpression gave rise to subtypes of cortical neurons, capable of integrating structurally into the injured brain, more rapidly than neurons produced by previous protocols. Functional characterization of the grafted hES-iNs and their impact on the balance between brain excitation and inhibition are now highly warranted. This new stem cell source should be considered when, in the future, the most suitable candidate will be selected for clinical translation.

## Graphical Abstract

**Graphical Abstract:**
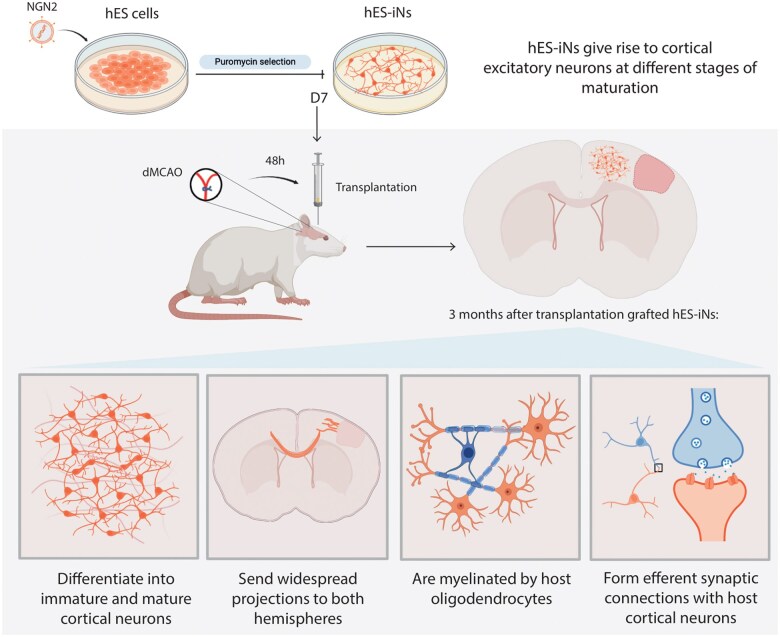

Significance statement Neuronal replacement through stem cell transplantation holds promise as a novel therapy for neurodegenerative diseases. Identifying the most suitable cell source for clinical translation will be essential. Here we report that human embryonic stem cells can be programmed to generate layer-specific cortical neurons. After transplantation, these neurons integrate rapidly into the stroke-injured adult rat cortex, establishing synaptic connections with host neurons and becoming myelinated by host oligodendrocytes. Our findings introduce a new stem cell source with potential use for neuronal replacement in human disorders affecting the cerebral cortex.

## Introduction

Ischemic stroke is characterized by the loss of neurons, leading to long-term sensory, motor, and cognitive impairments experienced by the majority of surviving patients.^1^ Effective treatments to improve function in the chronic phase after the insult are lacking. Stem cell-based approaches hold great promise as potential new therapies for human brain disorders. The clinical trials performed to date in ischemic stroke, involving the transplantation of mesenchymal stem cells, have not aimed at cellular replacement. Instead, the modest improvements have been attributed to the so-called bystander effect, that is, the stem cells’ trophic action and their ability to stimulate plasticity and modulate inflammation.^2–5^

Evidence from animal models indicates that stem cell transplantation may also induce functional improvement by replacing dead cells and reconstructing the stroke-injured brain. We have demonstrated that long-term neuroepithelial-like stem (lt-NES) cell-derived cortical neurons, produced from human induced pluripotent stem (hiPS) cells via neural induction and transplanted into stroke-injured adult rat cortex, establish afferent and efferent functional connections with host neurons in both hemispheres, improve neurological deficits, and regulate motor behavior 6 months after transplantation.^6–8^ The grafted lt-NES cells also give rise to mature oligodendrocytes capable of remyelinating host axons^9^. Similarly, human embryonic stem (hES) cell-derived visual cortical neurons, also generated via neural induction and implanted into the neurotoxin-injured mouse visual cortex, send axonal projections matching the normal ones and receive functional synaptic input from the host after 6 months.^10^ These studies have shown that grafted cells of human origin generated through neural induction take a long time to mature and become functionally integrated. Recently, Wu and collaborators have demonstrated that physical exercise could double the rate of neuronal integration of grafted human neural progenitor cells in the injured rat striatum.^11^ Additionally, cerebral organoids derived from hES cells and hiPS cells, when placed in the injury cavities or peri-infarct area of the adult rat visual cortex and primary motor cortex, established reciprocal connections with the host brain and responded to host stimuli within 2 and 6 months after transplantation, respectively.^12^^,^^13^ Taken together, these studies strongly support the notion that reconstruction of the injured adult brain is possible and raise the possibility that, in the future, this might be achievable also in a clinical setting. In further support of a possible clinical translation, cortically fated human lt-NES cells also differentiate to mature, functional cortical neurons^14^ and myelinating oligodendrocytes^9^ when transplanted onto organotypic cultures of the adult human cortex.

Identifying the most suitable human stem cell source is essential for translating experimental findings into clinical applications for brain repair in stroke patients. The ideal stem cells should be non-tumorigenic, exhibit proper functional characteristics after transplantation, and generate specific neuron subtypes. Here, we tested a protocol to differentiate hES cells via transcription factor (TF) programming to layer-specific functional cortical neurons.^15^ The objectives were 3-fold: (1) to characterize the hES-induced neurons (hES-iNs) *in vitro* prior to their transplantation; (2) to analyze the survival, phenotype, and projections of the grafted neurons 3 months after transplantation in a rat model of cortical ischemic stroke; (3) to assess whether the graft-derived axons establish synaptic contacts and are myelinated by host oligodendrocytes.

## Methods

For detailed information and protocols, see Supplementary material.

### hES-iN generation

hES-induced neurons were generated from WAO1 (H1) hES cells as previously described.^15^ Briefly, hES cells were cultured on Matrigel-coated 6-well plates in mTeSR medium. After reaching 80% confluence, cells were dissociated and replated on plates in mTeSR (StemCell Technologies, UK) medium supplemented with ROCK Inhibitor (10 µM, Y27632, Selleckchem). Twenty-four hours after seeding, the hES cells were transduced with TetO-Ngn2-T2A-Puro and FUW-M2rtTA lentiviruses. At day 0, mTeSR medium was replaced with an induction medium (DMEM/F12 supplemented with N2 [1:100, Thermo Fisher Scientific, Sweden] and B27 [1:50, Thermo Fisher Scientific, Sweden]), where doxycycline (2.5 μg/mL, Merck, Sweden) was added and kept until the end of the experiment to continuously induce NGN2 expression. From day 1 to 7, puromycin (1.25 μg/mL) selection was applied. Cells were collected on day 6 for RT-qPCR and on day 7 for transplantation. Cells subjected to immunostainings or electrophysiological recordings were transferred to coverslips on day 6 and analyzed 24 hours later.

### Distal middle cerebral artery occlusion and cell transplantation

Focal cortical ischemic injury was induced in adult male athymic, nude rats by distal middle cerebral artery occlusion (dMCAO), as described previously, with some modifications^8^ (details in Supplementary material). All procedures were conducted in accordance with European Union Directive 2010/63/EU and approved by the ethical committee for the use of laboratory animals at Lund University and the Swedish Department of Agriculture (Dnr. 5.8.18-07222/2021 M68-16).

### Immunocytochemistry and immunohistochemistry

A list of antibodies and detailed protocols for immunostainings, imaging, and quantifications are provided in the Supplementary material.

### RT-qPCR

RNA extraction from hES-iNs on day 6 of programming was performed with the RNeasy Mini Kit (QIAGEN) following the manufacturer’s protocol. TaqMan probes used are listed in the Supplementary material.

### Electrophysiology

Electrophysiological properties of hES-iNs were recorded on day 7^15^ using a HEKA double patch EPC10 amplifier (HEKA Elektronik, Lambrecht, Germany) and sampled at 10 KHz. Detailed protocol is provided in Supplementary material.

### iDISCO

Immunolabeling-enabled three-dimensional imaging of solvent-cleared organs (iDISCO) tissue clearing was performed according to Renier and collaborators^16^ with some modifications (details in Supplementary material). Briefly, animals were perfused and post-fixed in 2% paraformaldehyde for 1 hour and then kept in phosphate buffer saline (PBS) at 4 °C. Primary antibodies, mouse anti-STEM101 (1:500, Takara Bio Europe) and mouse anti-STEM121 (1:500, Takara Bio Europe), were added to identify human nucleus and processes, followed by incubation with the secondary antibody donkey anti-mouse Cy5. Clearing was performed using dibenzyl ether prior to sample imaging. Tissue-cleared samples were imaged in a sagittal orientation using a sCMOS-5.5-CL3 camera equipped light sheet microscope (Ultramicroscope II, LaVision Biotec, Germany) with a 2×/0.5 objective lens (MVPLAPO 2×) with a 6-mm working distance dipping cap. All imaging was performed using the Imspector-Pro219 software and scanned continuously with a step size of 10 μm at 3.2× magnification (7988 × 9472 pixels). Post-imaging visualization utilized the Arivis Vision 4D v.2.12.3 software.

### Immunoelectron microscopy

Rats were deeply anesthetized 3 months after transplantation with pentobarbital and transcardially perfused with 0.1 M PBS followed by ice-cold 2% formaldehyde, containing 0.2% glutaraldehyde, in 0.1 M PBS, pH 7.4. Brains were removed and then washed in 0.1 M PBS. Frontal 100-μm sections were cut on a Vibratome VT1000A (Leica, Germany), post-fixed in 1% osmium tetroxide in 0.1 M PBS, dehydrated in a graded series of ethanol and propylene oxide, and flat-embedded in Epon. Ultrathin sections were cut with a diamond knife. For post-embedding immunogold labeling of STEM121, ultrathin sections were incubated overnight in primary mouse anti-STEM121 antibody (1:500, Takara Bio Europe) at +4°C. A secondary antibody (goat anti-mouse IgG conjugated to 15-nm colloidal gold, EMS) diluted 1:20 in 0.1% BSA in PBS was added for 1.5 h and then washed with PBS. Sections were then fixed with 2% glutaraldehyde, washed with PBS, followed by distilled water, and stained with uranyl acetate and lead citrate. Ultrathin sections were examined and photographed using a transmission electron microscope JEM-100CX (JEOL, Japan).

### Statistical analysis

Statistical analysis was performed using Prism 9 software (GraphPad, Dotmatics). An unpaired t-test was used when data were normally distributed, and a Mann-Whitney U test when data did not pass the normality test. Significance was set at *P* < .05. Data are expressed as mean ± SEM.

## Results

### TF-programmed hES cells express markers for cortical excitatory neurons after 7 days of induction in vitro

We previously found that induction of NGN2 expression in hES cells gives rise to neuron-like cells by the end of the first week *in vitro.* After 2 weeks, these cells expressed the mature neuronal marker MAP2 and showed the morphology of mature neurons, exhibiting a pyramidal shape with rich arborization comparable to adult human cortical neurons.^15^ Here, we wanted to explore the potential use of hES-iNs for intracerebral transplantation. We first conducted a detailed *in vitro* evaluation of the differentiation level of the cells at the time point chosen for implantation (7 days), coinciding with the completion of the selection of transduced cells expressing the puromycin resistance gene.

**Figure 1.**
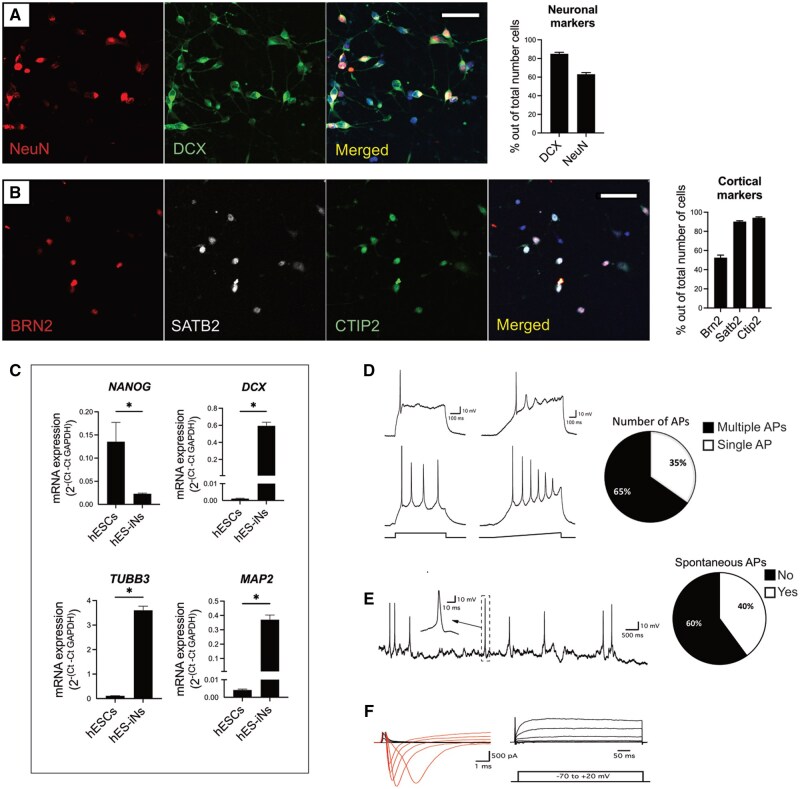
Overexpression of neurogenin 2 (NGN2) in hES cells generates immature and mature cortical neurons after 7 days of programming *in vitro*. **A-B**, Confocal images and quantifications of the expression of the neuronal progenitor marker DCX and the mature neuronal marker NeuN (**A**) and the upper and deep cortical layer markers BRN2 and SATB2; and CTIP2 (**B**), respectively, in hES-iNs at day 7 of programming. Scale bars, 50 μm. **C**, RT-qPCR analysis of gene expression (*NANOG*, *DCX*, *TUBB3,* and *MAP2*) in hES cells and hES-iNs after 6 days of programming. Data are presented as mean ± SEM (n = 3-4). Significance was set as p < 0.05. **D**, Whole-cell patch-clamp recordings from hES-iNs at day 7. Voltage traces illustrate the ability of the cells to generate single (upper traces) or multiple (lower traces) APs during a current step (left, 30 pA, and 20 pA, respectively) or ramp (right, 0-50 pA) injection. A pie chart shows the ratio of the cells with single (35%) or multiple (65%) APs upon depolarization challenge (n = 23). **E**, A voltage trace illustrating the ability of a portion of cells to spontaneously generate APs at RMP. A pie chart shows a ratio of the cells with (60%) or without (40%) spontaneous APs (n = 15). **F**, Current traces illustrating the inward sodium (red) and outward potassium (black) currents during voltage steps depolarization from -70 mV with 10 mV increments.

We observed that 84.9 ± 1.7% of the hES-iNs were immunopositive for the immature neuronal marker doublecortin (DCX) after 7 days *in vitro,* while the mature neuronal marker NeuN was expressed in 63.0 ± 1.8% (Figure 1A). Only DCX was expressed in 23.8 ± 4.9% of the cells, 61.0 ± 3.5% expressed both DCX and NeuN, while 2.0 ± 1.2% expressed only NeuN. The proliferation marker Ki67 was expressed in 6.1 ± 1.7% of the cells (Supplementary Figure 1A). We found no expression of the pluripotency marker Nanog, and just a few cells (<1%) were immunopositive for the multipotency marker Sox2 (data not shown), suggesting the presence of a small population of neural stem cells. Taken together, our data provide evidence that NGN2 programming of hES cells gives rise to about 87% of neurons at different stages of differentiation: immature neurons (DCX^+^/NeuN^−^), intermediate stage (DCX^+^/NeuN^+^), and a small population of mature neurons (DCX^−^/NeuN^+^).

Interestingly, we found that 95.0 ± 0.1% of the generated cells were immunopositive for KGA, a protein essential for differentiation of neural progenitor cells^17^ as well as a marker for excitatory neurons (Supplementary Figure 1B), suggesting that cells not expressing neuronal markers were already committed to become neurons. No cells expressed the marker for inhibitory neurons GAD65/67^+^ (data not shown). The hES-iNs expressed markers characteristic of both upper cortical layers, such as BRN2 (52.5 ± 2.6%) and SATB2 (90.1 ± 1.0%), and deep layers, for example, CTIP2 (94.1 ± 1.0%) (Figure 1B). Interestingly, and in line with our previous data^15^, the vast majority (93.8 ± 1.0%) of the cells co-expressed upper and deep cortical layer markers (Figure 1B). No expression of the astrocyte or oligodendrocyte markers GFAP and OLIG2, respectively, was found (data not shown).

The gene expression data analyzed by RT-qPCR were consistent with the immunocytochemical results. We found a decrease in the expression of *NANOG* and an increase of the neuronal markers *DCX*, *TUBB3* (Tuj1), and *MAP2* in cells programmed for 6 days compared to non-programmed ES cells (Figure 1C). We also observed increased expression of specific cortical markers such as *POU3F2* (BRN2; a marker of upper cortical layers) and *TBR1* (a marker of deep layers) (Supplementary Figure 1C).

Using whole-cell patch-clamp recordings, we found that already at 7 days, the hES-iNs exhibited all basic properties of functional neurons, that is, the ability to fire Action Potentials (APs) (Figure 1D-E) and presence of both fast inward sodium and sustained outward potassium currents (Figure 1F). The average resting membrane potential (RMP) of the recorded cells was −59.3 ± 1.55 mV. The majority of the cells (65%, 15 out of 23 in total) were able to generate multiple APs upon step or ramp current injection, while a smaller portion (35%, 8 out of 23 in total) only fired a single AP (Figure 1D). These observations indicate the occurrence of more and less mature neuronal populations simultaneously. We also observed spontaneous AP firing at RMP in 40% (6 out of 15 in total) of those with multiple APs (Figure 1E). When characterizing the basic parameters of APs (Supplementary Table 2), we observed that, on average, there were fast, high-amplitude APs with kinetics corresponding to a well-functioning neuron (AP amplitude = 58.26 ± 2.4 mV; AP threshold = −28.74 ± 1.01 mV; AP half duration = 2.85 ± 0.29 ms and afterhyperpolarization potential [AHP] = 13.17 ± 1.1 mV). However, when looking at passive membrane properties, the average input resistance (Ri) was 2 634 ± 212 MΩ, which is relatively high but typical for newly programmed, and still maturing cells with small somas and limited arborization complexity. No spontaneous synaptic activity was detected in any of the 23 recorded cells (from 7 coverslips across 2 different rounds of programming) at this time point. To summarize, the electrophysiological analysis suggests that although the hES-iNs have acquired certain neuronal characteristics, they are still young and not yet synaptically integrated at 7 days.

### TF-programmed hES cells differentiate into layer-specific cortical neurons after transplantation into stroke-injured rat cortex

We have previously shown that NGN2-induced hES-iNs placed onto slices of adult human cortex survived and differentiated to functional neurons with extended neurites at 4 weeks after transplantation. Some hES-iNs expressed the upper-layer cortical marker SATB2.^15^ Here, we wanted to determine the survival and differentiation of the NGN2-induced hES-iNs after intracortical transplantation in rats with cortical ischemic stroke.

Rats were subjected to dMCAO followed by MRI 24 h later to determine the volume and confirm the location of the lesion. The injured area was restricted to the somatosensory cortex in all animals, with an infarct volume of 8.6 ± 3.1 mm^3^. At 48 h post-dMCAO, hES-iNs programmed for 7 days were transplanted in close proximity to the damaged cortex, and animals were sacrificed 1 and 3 months later. Transplantation at 48 h was chosen based on the mild microglia activation and better cell survival at this time-point as compared to 1 week post-stroke.^18^^,^^19^

**Figure 2.**
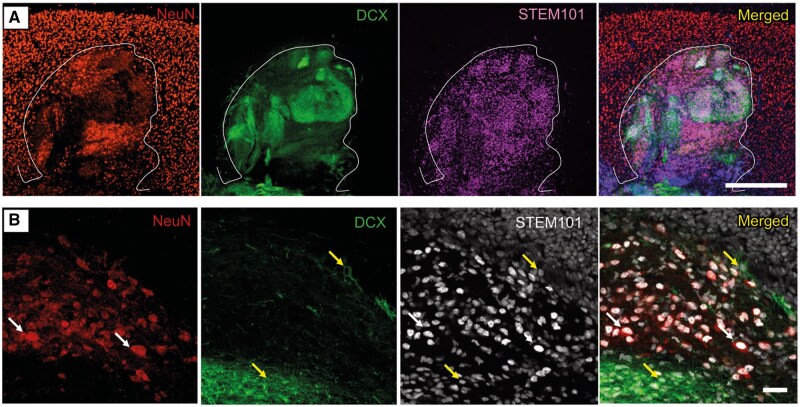
Grafted hES-iNs express markers of mature and immature neurons 3 months after transplantation into the stroke-damaged somatosensory cortex. **A-B**, Confocal images showing neuronal progenitors (DCX, green) and mature neurons (NeuN, red) in the transplant core (STEM101, white, human nuclear marker). **A**, Overview of grafted hES-iNs expressing NeuN, DCX and STEM101. Scale bar, 500 μm. **B**, High magnification confocal images of colocalization of NeuN and STEM101 (white arrows) and DCX and STEM101 (yellow arrows), respectively. Nuclear staining (Hoechst, blue) is included in the merged panel. Scale bars, 20 μm. n = 6.

At 3 months after transplantation, the vast majority of grafted cells, identified with the human nuclear protein marker STEM101, surrounded the lesion, mostly in the somatosensory cortex, with some cells in the M1 motor cortex. In the larger grafts, a substantial number of cells were also found in the corpus callosum (Supplementary Figure 2A). Grafted cells expressed markers of both immature and mature neurons (DCX and NeuN, respectively). The distribution of cells expressing these markers within the transplant core was heterogeneous. Some areas only expressed DCX (39.98 ± 4.3% of the total graft area), whereas others solely expressed NeuN (35.29 ± 3.1%) (Figure 2A-B). A minority of grafted cells co-expressed DCX and NeuN, suggesting that they were, at this time point after transplantation, still in an intermediate stage of maturation (Supplementary Figure 2B). Quantification of these double-positive cells was not possible due to the large number of cells in the graft and the cytoplasmic nature of DCX staining. In contrast to the findings at three months, the 1-month grafts predominantly expressed the immature neuronal marker DCX and only a few grafted cells expressed NeuN (Supplementary Figure 3A-B), demonstrating progressive maturation of the graft over time.

To assess the proliferative status of the graft, we stained for Ki67 and STEM101. While at 1-month post-transplantation, numerous Ki67-positive cells were observed within the graft (Supplementary Figure 3C), we distinguished two different patterns of Ki67 expression at 3 months after grafting. In most areas, we found homogenous, sparse expression of Ki67 in 0.9 ± 0.1% of the cells. We also detected highly proliferative islets with 9.6 ± 1.0% of cells being positive for Ki67. One or two islets were observed in all animals (Supplementary Figure 2C-D). Areas with high proliferation corresponded with the more immature areas, as observed by colocalization of Ki67 with DCX (Supplementary Figure 2E).

Fewer than 1% of the cells within the graft expressed markers for astrocytes (STEM123, corresponding to human-specific glial fibrillary acidic protein [GFAP]), and about 1% expressed the oligodendrocyte lineage marker OLIG2 (Supplementary Figure 4A-B).

Previous studies have shown that human stem cell-derived transplants increase endogenous oligodendrogenesis.^9^^,^^20^ We therefore assessed the effect of hES-iN transplantation on the number of endogenous oligodendrocytes 3 months after transplantation. Transplanted rats showed a significant increase in OLIG2^+^ cells in the middle part of the corpus callosum compared to stroke-subjected controls (Supplementary Figure 5A-B). These OLIG2^+^ cells did not express the human nuclear marker STEM101, demonstrating they were not of graft origin.

**Figure 3.**
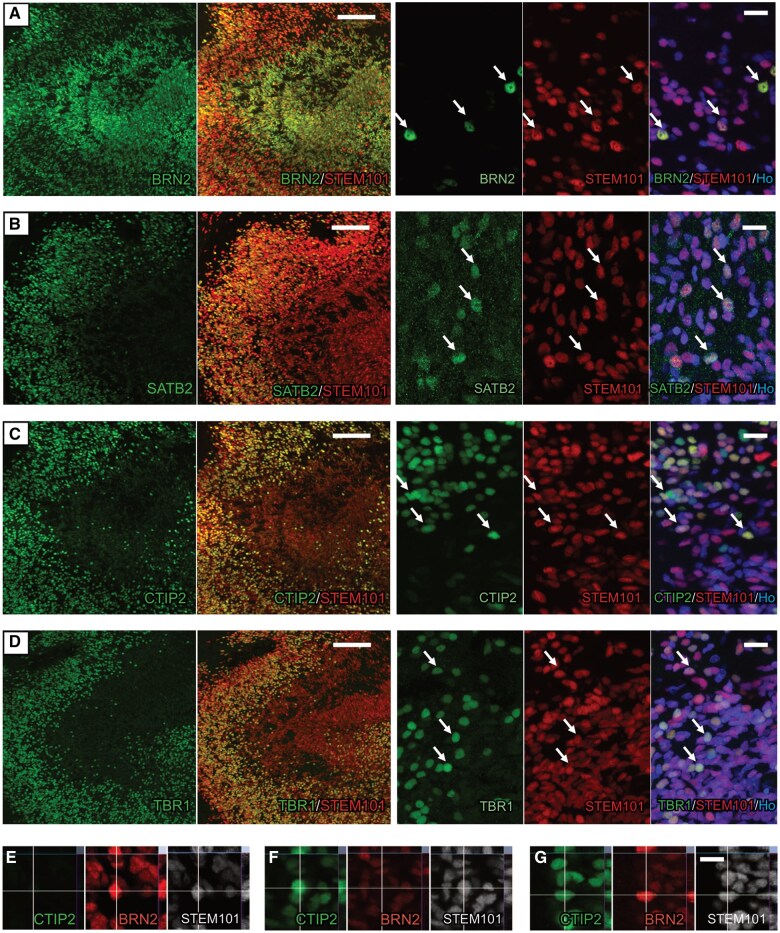
Grafted hES-iNs differentiate into layer-specific cortical neurons 3 months after transplantation into rat stroke-damaged somatosensory cortex. **A-D**, Confocal images showing co-expression of the human nuclear marker (STEM101^+^ cells, red) with the upper cortical layer markers BRN2 (**A**) and SATB2 (**B**); and deep cortical layer markers CTIP2 (**C**) and TBR1 (**D**) in the core of transplantation. **E-G**, High magnification confocal images showing grafted hES-iNs (STEM101) expressing: BRN2 (**E**), CTIP2 (**F**); or both transcription factors (**G**). Arrows indicate colocalization. Scale bar in A-D low magnification, 500 μm. Scale bar in A-D high magnification, 20 μm. n = 6. Scale bar in E-G, 10 µm.

Next, we analyzed whether the grafted cells expressed layer-specific cortical markers that recapitulate the architecture of the cerebral cortex. Three months after grafting, within the transplant core, hES-iNs (STEM101^+^ cells) were immunopositive for either upper cortical layer markers, such as BRN2 or SATB2 (Figure 3A-B, E), or deep cortical layer markers, like CTIP2 and TBR1 (Figure 3C-D, F). Interestingly, cells with the same cortical marker were arranged in layers and spatially separated within the graft (Figure 3A-D). In contrast to what we observed *in vitro*, only a few grafted hES-iNs co-expressed markers for upper and deep cortical layers (Figure 3G).

### Grafted, TF-programmed hES cells project widely, become myelinated, and form synapses with host neurons in the stroke-injured rat brain

**Figure 4.**
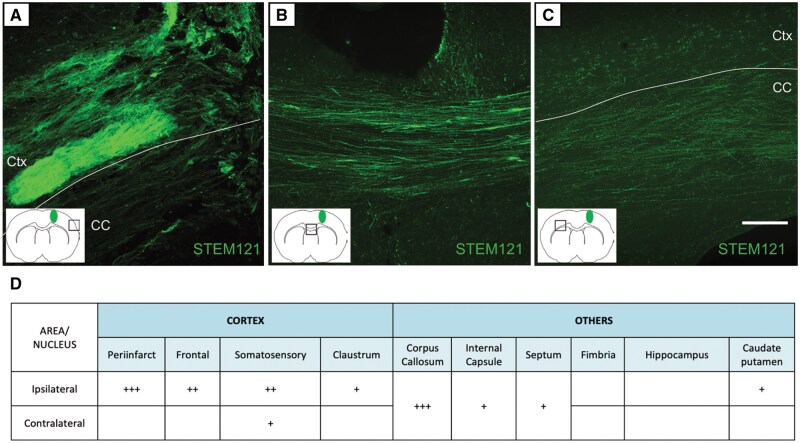
Intracortical grafts of hES-iNs send widespread axonal projections in the stroke-damaged rat brain. **A-C**, Presence of fibers expressing the human cytoplasmic marker STEM121 in different areas of the rat brain: peri-infarct area (**A**), corpus callosum (**B**) and contralateral somatosensory cortex (**C**). Scale bar, 100 μm. **D**, Semi-quantitative representation of the density of STEM121^+^ fibers derived from the graft: +, low density of 1-5 fibers; ++, medium density of up to 50 fibers; and +++, high density of more than 50 fibers. Ctx: cortex. CC: corpus callosum. n = 6.

We then analyzed the pattern of projections of the grafted hES-iNs using immunohistochemistry and iDISCO (Figure 4 and Supplementary Video 1). Three months after transplantation, many graft-derived fibers (stained by STEM121, a human cytoplasmic protein marker) reached the peri-infarct area (Figure 4A), and a substantial number were found in the ipsilateral frontal and somatosensory cortices. A high density of fibers was distributed throughout the corpus callosum (Figure 4B), and a few of them reached the contralateral somatosensory cortex (Figure 4C). Few fibers were detected in the caudate-putamen, septum, and internal capsule.

**Figure 5.**
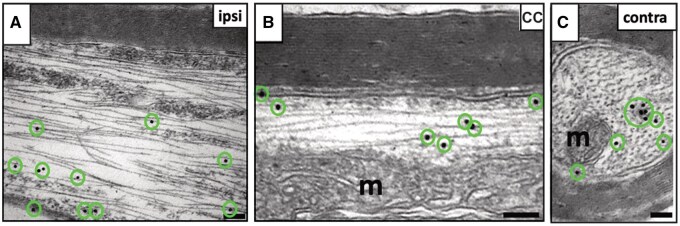
Host oligodendrocytes myelinate grafted hES-iNs-derived axons in the stroke-damaged rat brain. **A-C**, Representative iEM images of STEM121^+^ hES-iN-derived axons myelinated by host oligodendrocytes in ipsilateral somatosensory cortex (ipsi, **A**) corpus callosum (CC, **B**) and contralateral somatosensory cortex (contra, **C**). Note: Gold particles are marked with circles; m, mitochondrion. Scale bars, 0.1 μm. n = 3.

To determine if the axons derived from the grafted hES-iNs had become myelinated and established synaptic connections with host cells, we performed post-embedding immunogold labeling of STEM121 on ultrathin sections. Immunoelectron microscopy (iEM) analysis demonstrated the presence of individual 15-nm gold particles in STEM121^+^ axons and axon terminals. We observed STEM121^+^ hES-iN-derived axons in the corpus callosum and in the ipsilateral and contralateral cortices (Figure 5A-C). Human STEM121^+^ axons exhibited ultrastructural features similar to those of host axons, such as microtubules, neurofilaments, and mitochondria, and were enclosed by compact myelin sheaths originating from STEM121^-^ host oligodendrocytes (Figure 5A-C).

**Figure 6.**
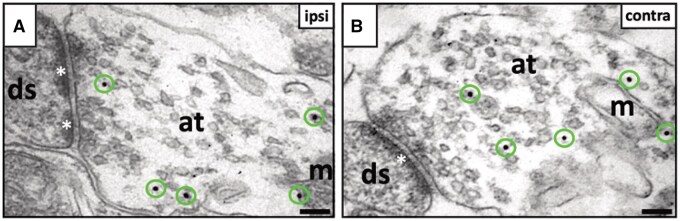
Grafted hES-iNs establish functional synapses with host neurons in both hemispheres of the stroke-injured rat brain. **A-B**, Asymmetric synapses with perforated (asterisks in **A**) and continuous, non-perforated postsynaptic densities (asterisk in **B**) in host dendritic spines (ds) connected with grafted hES-iN-derived presynaptic axon terminals (at). Note: Gold particles are marked with circles; m, mitochondrion. Scale bars, 0.1 μm. n = 3.

The hES-iN-derived axon terminals had established characteristic synapses with host dendritic spines in the ipsi- (Figure 6A) and contralateral (Figure 6B) cortex. These STEM121^+^ synaptic contacts had vesicles in the presynaptic terminal, a synaptic cleft, and a postsynaptic membrane with postsynaptic densities. The vast majority (91.7%) of postsynaptic densities were ­continuous non-perforated (Figure 6B), and only 8.3% were perforated (Figure 6A). Most (89.4%) STEM121^+^ synaptic contacts were axodendritic and displayed the ultrastructural characteristics of asymmetric excitatory/glutamatergic synapses, for example, prominent postsynaptic density, a wide synaptic cleft, and spherical synaptic vesicles. The STEM121^+^ presynaptic terminals had docked synaptic vesicles at the presynaptic membrane (Figure 6), indicating the functional activity of synapses.^21^

## Discussion

Here, we show for the first time that subtype-specific human cortical neurons, which mature and integrate rapidly into host neural circuitry after transplantation in the stroke-injured rat brain, can be generated by NGN2-programming of hES cells. Already following a 7-day programming protocol *in vitro*, the hES cells had given rise to excitatory neurons at different stages of maturation, co-expressing both upper and deep cortical layer markers. As early as 3 months after intracortical grafting, the hES-iNs had matured to layer-specific cortical neurons, extended widespread projections to different regions of the host brain, become myelinated by host oligodendrocytes, and established efferent synaptic connections with host neurons.

In a clinical study,^22^ we observed that lesions in the cerebral cortex are associated with long-lasting neurological deficits in the majority of stroke patients. Successful replacement of the cortical neurons lost after stroke could, therefore, have a major impact on the magnitude of functional recovery. Two main strategies have been used to generate cortical neurons from human pluripotent stem cells (hES and hiPS cells): *First*, through an initial neural induction via dual SMAD and WNT inhibition using small molecules.^14^^,^^23^^,^^24^ We have previously shown that hiPS cell-derived lt-NES cells, following a cortical priming protocol, generate neurons expressing markers of both upper and deep layers after 8 weeks in culture.^14^ Similarly, using ES cells, the deep-layer cortical marker TBR1 was expressed *in vitro* at 3 weeks, while 2–3 months were needed for expression of the upper layer cortical markers BRN2 and SATB2.^23^^,^^24^  *Second*, through cell conversion driven by forced expression of defined TFs. The *in vitro* overexpression of the TF NGN2 in hES and hiPS cells cocultured with mouse glia was shown to produce 95% mature excitatory neurons at day 21. However, only the expression of the upper layer cortical markers BRN2 and CUX1 was reported at this time point.^25–27^ An improved protocol using a combination of NGN2 overexpression and neural induction, also in the presence of mouse glia, gave rise to the expression of the upper cortical markers BRN2, CUX1, and SATB2 at day 21, but no deep-layer cortical markers such as CTIP2.^28^

We report here, using a similar protocol with overexpression of NGN2 in hES cells but without mouse glia or neural induction, that about 95% of the cells expressed a marker for excitatory neurons already at day 7 *in vitro*. Co-expression of markers from both upper and deep cortical layers was also found in 95% of the hES-iNs at this time point. In contrast, previous protocols involving overexpression of NGN2 required 3 weeks to generate primarily upper layer cortical neurons.^25^ This discrepancy could be explained by differences in the generation of hES-iNs compared to other protocols,^15^^,^^25^ for example, whether mouse glia is added, and the timing of their addition. It should be pointed out that single-cell data from 8-week hES-iNs cocultured with mouse glia reveal molecular characteristics typical of human fetal cortical neurons, such as expression of both upper and deep cortical layer markers.^15^ Analogous to observations in mouse neural precursors,^29^ the transcriptomic profile of hES-iNs shows molecular priming toward cortical neurons of diverse subtypes.

Regarding functional maturation, protocols involving neural induction have been reported to take up to 2 months to generate cells able to fire multiple APs,^24^ and the combination of NGN2 overexpression with neural induction takes 14 days.^28^ Using our protocol, 65% of the NGN2-programmed hES-iNs generated multiple APs already on day 7. Taken together, these data indicate that our protocol to produce human cortical neurons from pluripotent stem cells *in vitro* is the fastest one in terms of both neuronal maturation and functionality.

Interestingly, we found that the hES-iNs expressed SATB2, the primary marker of transcallosal projection neurons (TCPNs), as early as day 7 *in vitro*. In contrast, previous protocols for generating cortical neurons have typically required from 21 up to 100 days to produce SATB2-positive neurons.^23^^,^^24^ Hypothetically, reconstructing transcallosal pathways and restoring interhemispheric communication by transplantation of neurons rich in TCPNs could be particularly important for functional recovery after stroke.^8^^,^^30^

We provide evidence for the fast maturation of NGN2-programmed hES cells to cortical neurons, also in intracerebral grafts. Our transplantation study is the first one performed with cortical neurons generated via TF programming. It has been reported, though, that human pluripotent stem cell-­derived cortical progenitors, generated via neural induction and grafted into the injured adult visual or somatosensory rodent cortex, form mainly immature and proliferative grafts at 2 months post-transplantation (with about 80% of cells expressing DCX and only about 10% expressing NeuN). Some grafts contained rosette-like structures, similar to the areas of high proliferation found in our grafts.^7^^,^^10^ In line with these findings, hES-iNs grafts at 1 month post-transplantation consisted predominantly of immature neurons, while by 3 months we observed regions within the grafts with different degrees of maturation, but only 40% of the grafts were composed of immature neurons and as much as 35% of mature neurons.

The persistence of immature neurons within the grafts would conceivably limit their contribution to circuit repair. Importantly, though, it has been shown with other cell sources, such as cortically primed lt-NES cells, that grafts continue to mature over time. At 2 months after transplantation, around 80% of the cortically primed lt-NES cells expressed the immature neuronal marker DCX and 13% the mature neuronal marker NeuN.^7^ With time, as observed at 6 months after transplantation, only 3% of the grafted cells expressed DCX and 41% expressed NeuN.^8^

The expression pattern of different cortical markers within the hES-iN grafts further illustrates their rapid maturation. We observed many grafted cells expressing markers of either upper or deep cortical layers organized in distinct clusters, suggesting some level of organization. Interestingly, we have previously shown that individual NGN2-programmed hES-iNs co-expressed both deep and upper cortical layer markers after 2 months *in vitro.*^15^ In contrast, we found here that the grafted cells expressed cortical markers in a layer-specific manner. Our findings raise the possibility that the lack of generation of specific cortical layer phenotypes *in vitro* after TF programming could be due to the absence of signaling molecules and growth factors present in the *in vivo* environment.

It is noteworthy that grafts of hiPS cell-derived cortical progenitors, generated via neural induction and transplanted into healthy mouse cortex, showed clear segregation of deep and upper cortical layers only at 5 months, while at 3 months, no obvious cytoarchitecture was observed.^31^ On the other side, 6 months after transplantation of hES or hiPS cell-derived cortical progenitors into the injured cortex of adult mice or rats, both upper and deep cortical layer neurons were found. However, no segregation of cortical neurons was observed in this case.^7^^,^^8^^,^^10^

Our observations with hES-iNs grafts regarding integration into host circuitry, including myelination and synapse formation at 3 months, are comparable to those with grafts generated through neural induction protocols at 6-8 months.^8^^,^^10^ The differences observed in maturation speed between TF programming and neural induction may, hypothetically, be explained by the extent to which each method mimics normal brain development. Neural induction guides the cells through stages resembling embryonic cortical development, in which deeper layer neurons form first, leading to a slow maturation process. In contrast, TF programming directly pushes the cells into a mature state, skipping part of the intermediate processes.^32^ Consistent with previous studies using transplantation of human pluripotent stem cell-derived cortical neurons into stroke-injured brains,^8^^,^^33^ the grafted hES-iNs extended axonal projections to both ipsilateral and contralateral hemispheres. The projection patterns of hES-iNs at 3 months closely resembled those from cortically primed lt-NES cells at 6 months after transplantation (Palma-Tortosa 2020).^8^

Here, we have introduced a new human-derived neural cell source, generated from pluripotent stem cells, that exhibits structural integration into host neuronal circuitries after transplantation into the stroke-injured rat brain. Our findings provide evidence that intracortical grafts, generated by TF overexpression in hES cells, exhibit faster and more organized cortical maturation as compared to grafts derived from human pluripotent stem cells using small molecule differentiation protocols. It is noteworthy that the TF programming here generated almost exclusively cortical neurons. In contrast, differentiation using small molecules can give rise not only to neurons but also to other cell types, such as oligodendrocytes, essential for the regeneration of stroke-injured brains.^9^ The lack of myelinating cells in grafts of hES-iNs could be solved by transplanting these cells together with stem cell-derived oligodendrocytes to supply the injured brain with both cell populations. Behavioral assessments and functional characterization will be essential to evaluate the usefulness of the present protocol and the generated hES-iNs for clinical translation. A current advantage of pluripotent stem cells in a clinical translational perspective is the availability of human pluripotent stem cell banks and GMP-grade lines, which are already used in ongoing clinical trials.^34–36^ Significant work remains to select the optimal source for the generation of the specific neurons and glia needed to effectively reconstruct stroke lesions and achieve maximum functional recovery.

## Supplementary Material

sxaf049_Supplementary_Data

## Data Availability

The data supporting the findings of this study are contained within the manuscript or available from the corresponding authors on reasonable request.
