# Supplementary material for: Human cortical neurons rapidly generated by embryonic stem cell programming integrate into the stroke-injured rat cortex
Source: Stem Cells. 2025 Jul 12;43(11):sxaf049. doi: 10.1093/stmcls/sxaf049 (PMC12532315; doi:10.1093/stmcls/sxaf049)
Supplement: sxaf049_Supplementary_Data [file sxaf049_supplementary_data.zip › Supplementary material_Martinez-Curiel Stem Cells Final version.docx]

**SUPPLEMENTARY MATERIAL**

**Human cortical neurons rapidly generated by embryonic stem cell programming integrate into the stroke-injured rat cortex**

Raquel Martinez-Curiel, Mazin Hajy, Oleg Tsupykov, Linda Jansson, Natalia Avaliani, Juliane Tampé, Emanuela Monni, Galyna Skibo, Olle Lindvall, Sara Palma-Tortosa and Zaal Kokaia.

**METHODS**

**Animals**

Twelve athymic nude rats (Crl: NIH-Foxn1 RNU), 8 weeks of age, were purchased from Charles River Laboratories. The animals were housed in individually ventilated cages under standard temperature and humidity conditions and a 12 h light/dark cycle with free access to food and water.

The sample size was estimated based on our previous studies^1^.

**Distal middle cerebral artery occlusion and cell transplantation**

For induction of cortical stroke, animals were anesthetized with isoflurane (3.0% induction; 1.5% maintenance) mixed with air, exposing the temporal bone. A craniotomy of 3 mm was made, the *dura mater* was carefully opened, and the cortical branch of the middle cerebral artery was ligated permanently by suture, cauterized, and cut. Both common carotid arteries were isolated and ligated for 30 min. After releasing the common carotid arteries, surgical wounds were closed.

Stroke analysis was carried out by magnetic resonance imaging (MRI) 24 h after experimental ischemia using the Biospec Avance III, 9.4T (BrukerBioSciences Corporation, Ettlingen, Germany). Animals with no ischemic lesion are excluded from the study.

Intracortical transplantation of hES-iNs was performed stereotactically 48 h after dMCAO as described previously.^1^ Briefly, on the day of surgery, programmed hES-iNs on day 7 were resuspended to a final concentration of 100.000 cells/μL in cytocon buffer. A volume of 1 μL was injected in 3 sites at the following coordinates (from bregma and brain surface): anterior/posterior: +1.5 mm; medial/lateral: +2.0 mm; dorsal/ventral: −2.0 mm; and anterior/posterior: +0.5 mm; medial/lateral: +1.5 mm; dorsal/ventral: −2.0 mm and anterior/posterior: +0.5 mm; medial/lateral: +2.5 mm; dorsal/ventral: −2.5 mm.

**Immunocytochemistry and immunohistochemistry**

hES-iNs plated on glass coverslips were fixed on day 8 in 4% paraformaldehyde (Sigma) for 20 min at room temperature. Cells were permeabilized with 0.025% Triton X-100 in 0.1 M potassium phosphate buffered saline (KPBS) and blocked with 5% of normal donkey serum (NDS) for 45 min. Primary antibodies (Supplementary Table 1) were diluted in blocking solution and applied overnight at 4ºC followed by 3 rinses with KPBS. Fluorophore-conjugated secondary antibodies (Supplementary Table 1) (1:500, Jackson Immunoresearch, UK) diluted in blocking solution were applied for 2 h at room temperature. Afterward, cells were rinsed 3 times with KPBS, and nuclei were stained with Hoechst (Molecular Probes) for 10 min in KPBS at room temperature. Stained glass coverslips were mounted on microscopy slides with a mounting medium containing Dabco (Merck).

Immunohistochemistry in rat slices was done as follows: 1 and 3 months after transplantation, animals were perfused transcardially with 4% PFA. The stored sections (30 μm) were rinsed 3 times with KPBS and incubated in a blocking solution for 1 h (0.25 Triton X-100 in 0.1 M KPBS [TKPBS] with 10% NDS). The rest of the procedure for cell cultures follows the procedure described above.

Some stainings required antigen retrieval (see Supplementary Table 1) before the permeabilization step. Cells and rat sections were incubated with sodium citrate, pH 6.0, Tween 0.05% for 30 min at 65ºC.

**Microscopical analysis and quantification**

*In vitro* quantifications were performed using 20x images taken using a confocal microscope (LSM 780, Zeiss, Germany). Using Image J software, several positive cells were counted by sampling various regions from different coverslips. The total number of cells in each region of interest was counted using Hoechst staining.

Overview images of rat brain slices stained with STEM101, NeuN, DCX, and Ki67 were taken using a Virtual Slide Scanning System (VS-120-S6-W, Olympus, Germany).

The volume of infarction was measured in sections stained with NeuN. The intact area was determined by cells in both the ipsilateral and contralateral hemispheres, marked out, and measured using ImageJ software. The infarcted area was determined by subtracting the non-lesioned (NeuN-stained) area in the damaged hemisphere from the corresponding location in the contralateral hemisphere. The lesion volume was calculated by multiplying the infarcted area by the thickness and spacing between sections (300 μm).

The graft area was determined using sections stained with STEM101. The immature and mature regions within the graft were determined by outlining areas positive for DCX and NeuN, respectively. Co-expression was assessed via confocal microscopy by observing the overlap of the two chosen markers within the same plane and area. Co-expression of layer-specific cortical markers (BRN2, CTIP2) with the human nuclear marker (STEM101) was performed using 63x confocal images.

Quantification of OLIG2^+^ cells in the corpus callosum in rat slices was performed in 20x confocal images (10 μm thick z stack).

Evaluation of areas reached by hES-iNs-derived fibers was performed using a Virtual Slide Scanning System (VS-120-S6-W, Olympus, Germany). Fiber density was assessed semi-quantitatively in 10 μm-thick maximum intensity projection confocal images captured with a 63x objective. Three to five images were analyzed for each area.

**RT-qPCR**

After extraction, RNA purity and concentration were determined using a NanoDrop spectrophotometer (ND-1000). RNA (1 mg) was used for cDNA synthesis with qScript cDNA SuperMix (QuantaBio). TaqMan probes (Thermo Fisher Scientific, Sweden; NANOG Hs02387400_g1, HS00300164_s1; DCX, Hs00167057_m1; BIIITUB, Hs00964963_g1; MAP2, Hs00258900_g1; POU3F2, Hs00271595_s1; and TBR1 Hs00232429_m1) were used and RT-qPCR was run in triplicate samples on a QuantStudio™ 1 Real-Time PCR System (Thermo Fisher Scientific, Sweden) with GAPDH as the housekeeping gene (HS02786624_g1)

**Electrophysiology**

Electrophysiological recordings were done with HEKA double patch clamp EPC10 amplifier using PatchMaster for data acquisition. For whole-cell patch-clamp recordings, coverslips were transferred to the recording chamber and constantly perfused with carbonated artificial cerebrospinal fluid (ACSF) containing (in mM): 119 NaCl, 2.5 KCl, 1.3 MgSO4, 2.5 CaCl2, 26 NaHCO3, 1.25 NaH2PO4, and 11 glucose (pH ~7.4, osmolarity ~305 mOsm). Patch pipette was filled with an internal solution containing (in mM): Kgluconate 122.5, KCl 17.5, NaCl 8, KOH-HEPES 10, KOH-EGTA 0.2, MgATP 2, and Na3GTP 0.3 (pH ~7.2, 295 mOsm). The average pipette tip resistance was ~4-5 MW, and recordings were done at 32ºC. Pipette current was corrected online before gigaseal formation. In contrast, fast capacitive currents were compensated for during cell-attached configuration.

Resting membrane potential (RMP) was measured in current clamp mode at 0 pA immediately after establishing whole-cell configuration. Input resistance (Ri) was calculated from a 5 mV pulse and monitored throughout the experiment. The ability to generate an action potential (AP) was determined by 500 ms square depolarizing current step injections at RMP, with 10 pA increments and ramp injection of 1 s depolarizing current, which was also used to determine the action potential threshold. AP amplitude was measured from threshold to peak, the half AP amplitude width was defined as the time between the rising and decaying phase of the AP measured at half the amplitude of the AP, and the afterhyperpolarization (AHP) amplitude was determined as the difference between the AHP peak and the AP threshold. Whole-cell sodium and potassium currents were observed in voltage-clamp mode at a holding potential of −70 mV, and 200 ms voltage steps were delivered in 10 mV increments.

**iDISCO**

Animals were perfused 3 months after transplantation and brains are post-fixed in 2% PFA for one hour, then kept in PBS at 4ºC shortly before the protocol started. Brains were washed three times in PBS for 30 min at room temperature (RT). Afterward, alcohol dehydration was performed in methanol/PBS with progressively higher methanol concentrations. The concentrations of methanol used were 20, 40, 60, 80, and 100%, and each dehydration step was performed for 30 min at RT. A last 100% methanol incubation was done for 1 h. The samples were then incubated overnight in 66% dichloromethane (DCM) and 33% methanol at RT, washed twice in 100% methanol for 1 h, and then kept overnight at 4ºC. On day 3, brains were incubated in freshly prepared 5% hydrogen peroxide overnight at 4°C. Following overnight incubation, the samples were rehydrated in methanol/0.2%/TritonX-100 in PBS with progressively lower methanol concentration. The concentrations of methanol used were 80, 60, 40, and 20% methanol, and finally, 0.2% TritonX-100+PBS, with each rehydration step being performed for 30 min. After that, two washes for one hour were done in 0.05% sodium azide and 0.2% TritonX-100 in PBS at RT with shaking. Following the washings, the samples were incubated in 0,05% sodium azide, 20% dimethyl sulfoxide (DMSO, Merk, Sweden), 0.2% TritonX-100, and 0.3 M glycine in PBS for 5 days at 37ºC. On day 9, two washings with 0.05% sodium azide, 0.2% TritonX-100, and 10 μg/mL heparin in PBS were done for 30 min at RT. Samples were then incubated with 0.05% sodium azide, 0.2% TritonX-100, 10% DMSO, and 6% of normal donkey serum (NDS) in PBS for 10 days at 37ºC. On day 19, samples were washed two times for half an hour with 0.05% sodium azide, 0.2% TritonX-100, and 10 mg/mL heparin in PBS at RT. After the washings, brains were incubated in a filtered solution containing the primary antibodies SC101 (Mouse 1:500, Stem Cells Inc) and SC121 (Mouse 1:500, Stem Cells Inc) diluted in 0.05% sodium azide, 5% DMSO, and 3% NDS in 0.2% Tween-20 and 10 mg/mL heparin in PBS, for 10 days at 37ºC. On day 29, the samples were washed 16 times for 25 min with 0.05% sodium azide, 0.2% Tween 20, and 10 mg/mL at RT. Following the washings, the brains were incubated in a filtered solution containing the secondary antibodies (Cy5, donkey anti-mouse, 1:500) diluted in 0.05% sodium azide and 3% NDS in 0.2 Tween-20 and 10 mg/mL heparin in PBS for 10 days at 37ºC. On day 39, the samples were washed 16 times for 15 min with 0.05% sodium azide, 0.2% Tween 20, and 10 mg/mL at RT. After washing, a methanol/PBS dehydration process was performed, and the samples were incubated overnight with DCM and methanol. On day 40, brains were incubated twice in DCM for 25 min and then in dibenzylether (DBE, Merk, Sweden) until the samples became clear. Before microscope analysis, samples were stored in DBE at RT with no light or air exposure.

**SUPPLEMENTARY FIGURES AND TABLES**

**
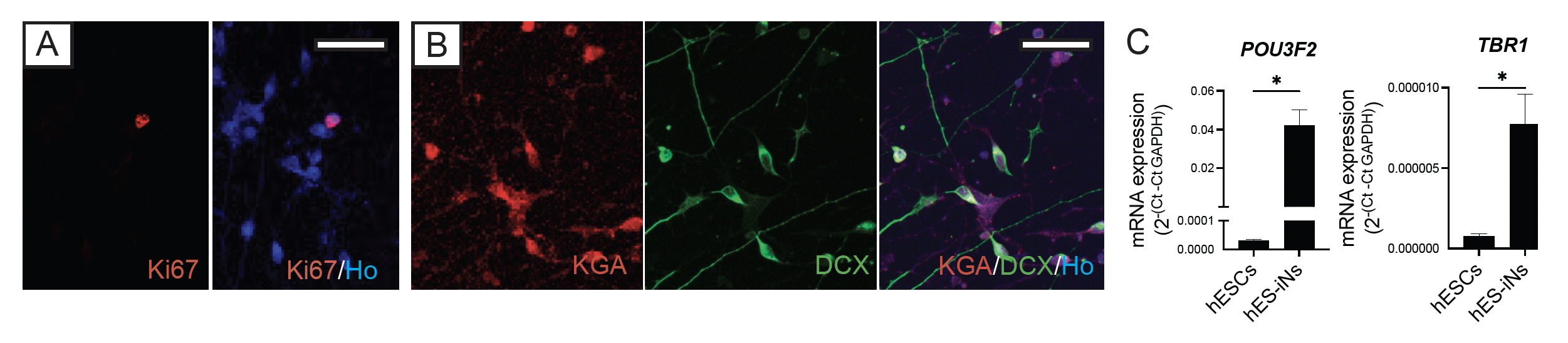
**

**Supplementary Figure 1**. ***In vitro* programming of hES cells gives rise to glutamatergic cortical neurons**. **A, B**, Representative confocal images showing the proliferation marker Ki67 (**A**), the glutamatergic marker KGA and the immature neuronal marker DCX (**B**). Scale bar, 50 µm. Ho: Hoechst, blue. **C,** Gene expression of *POU3F2* and *TBR1* in hES-iNs after 6 days of programming. Data are presented as mean ± SEM. Significance was set as p < 0.05.

**
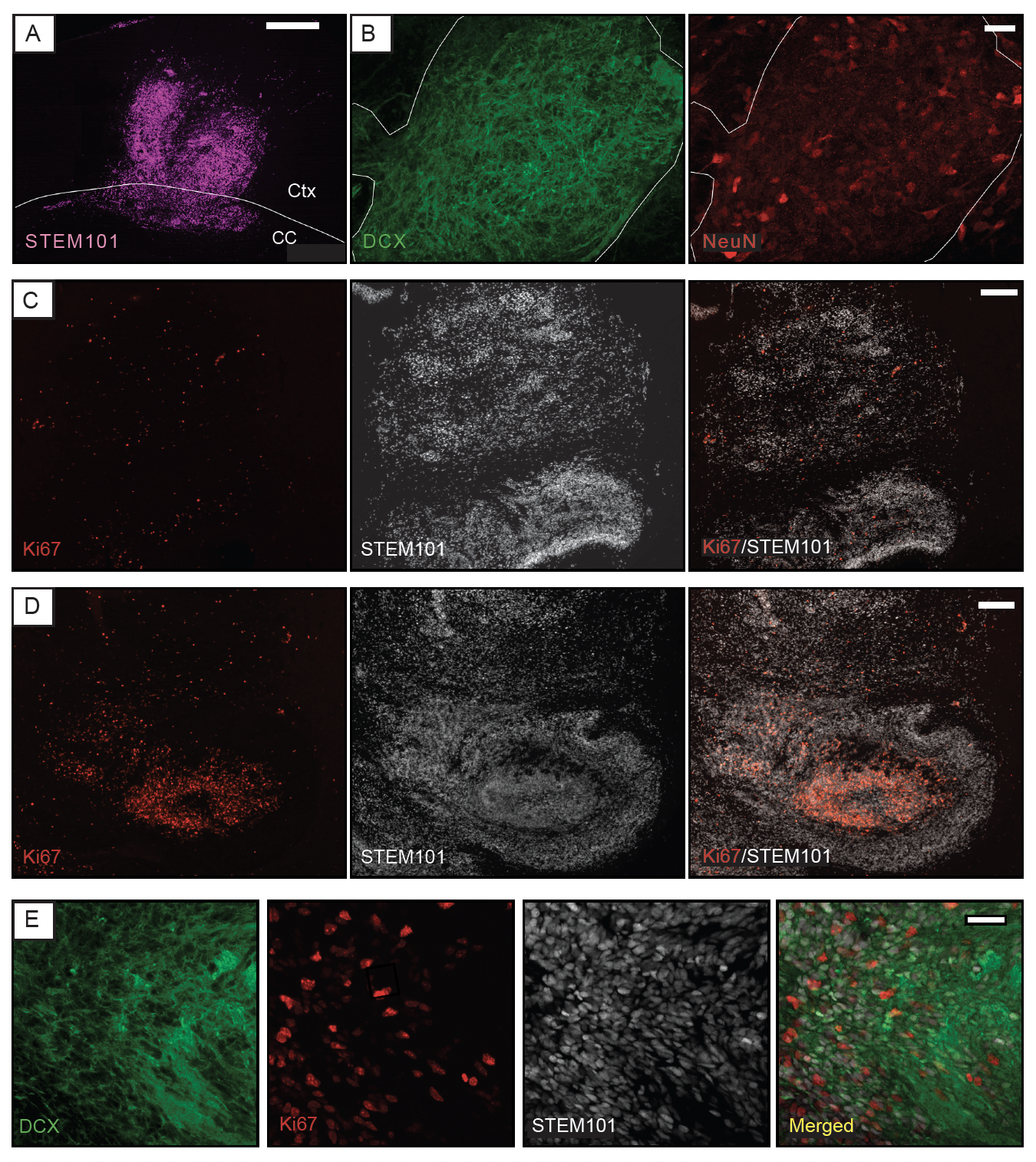
**

**Supplementary Figure 2**. **Intracortical transplantation of hES-iNs gives rise to heterogeneous grafts in terms of location and proliferation**. **A**, Overview of a large graft, identified by STEM101, showing grafted hES-iNs in the cortex and corpus callosum 3 months after transplantation in the injured-stroke cortex. Scale bar, 500 μm. **B**, hES-iNs show areas of colocalization of the immature neuronal marker DCX and mature neuronal marker NeuN (white outline). Scale bar, 20 μm. **C-D**, Proliferative grafted hES-iNs shown by human nuclear marker STEM101 and proliferation marker Ki67. **C**, Graft with sparse grafted proliferative cells. **D**, Grafted hES-iNs showed higher proliferation in less mature areas identified by rosette morphology. Scale bar, 200 μm. **E**, Representative confocal image showing the immature neuronal marker DCX and the proliferation marker Ki67 colocalizing with human nuclear marker STEM101. Scale bar, 20 μm. n=6.

**Supplementary Figure 3. Grafted hES-iNs remain as immature neurons and have a high proliferation rate 1 month after transplantation into the stroke-damaged somatosensory cortex.** Confocal images showing grafted cells (human nuclear marker, STEM101) colocalizing with: **A**, the immature neuronal marker DCX; **B**, the mature neuronal marker NeuN, and **C**, the proliferation marker Ki67. Scale bar in A-B, 50 μm, scale bar in C, 100 μm. n=5.

**Supplementary Figure 4**. **Grafted hES-iNs give rise to a small population of oligodendrocytes and astrocytes 3 months after transplantation in the injured-stroke cortex**. **A-B**, Representative confocal images showing graft-derived oligodendrocytes, shown by lineage oligodendrocyte marker OLIG2 (**A**), and graft-derived astrocytes, shown by specific human GFAP marker, STEM123 (**B**). Arrows indicate colocalization. Scale bar, 20 μm. n=6.

**Supplementary Figure 5. Intracortical transplantation of hES-iNs increases endogenous oligodendrogenesis in stroke-injured rat brains**. **A-B**, Representative confocal images (**A**) and cell quantification (**B**) of OLIG2^+^ cells in the middle part of the corpus callosum. Scale bar, 50 μm. Significance was set at p < 0.05. n=6.

| **Antibody** | **Host species** | ***In vitro*** | ***In vivo*** | **Notes** | **Company** |
| --- | --- | --- | --- | --- | --- |
| Primary antibodies | | | | | |
| BRN2 | Goat | 1:400 | 1:400 | AR | Santa Cruz |
| CTIP2 | Rabbit | 1:100 | 1:200 | AR | Merck |
| DCX | Goat | 1:400 | 1:400 |  | Santa Cruz |
| KGA | Rabbit | 1:1000 | - |  | Abcam |
| Ki67 | Rabbit | 1:500 | 1:250 |  | Abcam |
| NANOG | Rabbit | 1:150 | - |  | Abcam |
| NeuN | Rabbit | 1:500 | 1:500 |  | Abcam |
| OLIG2 | Rabbit | 1:500 | 1:500 |  | Abcam |
| SATB2 | Mouse | 1:100 | - | AR | Abcam |
| SATB2 | Rabbit | - | 1:200 | AR | Abcam |
| STEM101 | Mouse | - | 1:500 |  | Takara |
| STEM121 | Mouse | - | 1:500 |  | Takara |
| STEM123 | Mouse | - | 1:2000 |  | Takara |
| SOX2 | Rabbit | 1:200 | 1:200 |  | Merck Millipore |
| TBR1 | Rabbit | - | 1:300 |  | Gifted |
| Secondary Antibodies | | | | | |
| 488 anti-Goat | Donkey | 1:500 | 1:500 |  | Jackson ImmunoResearch |
| 488 anti-Mouse | Donkey | 1:500 | 1:500 |  | Jackson ImmunoResearch |
| 488 anti-Rabbit | Donkey | 1:500 | 1:500 |  | Jackson ImmunoResearch |
| Cy3 anti-Goat | Donkey | 1:500 | - |  | Jackson ImmunoResearch |
| Cy3 anti-Mouse | Donkey | 1:500 | 1:500 |  | Jackson ImmunoResearch |
| Cy3 anti-Rabbit | Donkey | 1:500 | 1:500 |  | Jackson ImmunoResearch |
| 647 anti-Mouse | Donkey | 1:500 | 1:500 |  | Jackson ImmunoResearch |
| 647 anti-Rabbit | Donkey | 1:500 | 1:500 |  | Jackson ImmunoResearch |

**Supplementary Table 1.** List of primary and secondary antibodies. **Related to section: *Immunostainings and quantifications***.

| Ri (MΩ) | 2634 ± 212 |
| --- | --- |
| RMP (mV) | -59.3 ± 1.5 |
| AP Ampliture (mV) | 58.3 ± 2.4 |
| AP Threshold (mV) | -28.74 ± 1.01 |
| AP AHP (mV) | 13.2 ± 1.1 |
| AP ½ duration (ms) | 2.85 ± 0.29 |

**Supplementary Table 2.** Table summarising passive membrane properties (Input resistance - Ri and RMP) and AP characteristics of the recorded cells (n=23).

**VIDEO FILES**

**Supplementary Video 1. Grafted hES-iN send widespread projections to different brain regions in 3 months after transplantation into the injured rat brain.** Grafted cells were immunostained with human nuclear marker STEM101 and human cytoplasmic marker STEM121. n=3.

**REFERENCES**

1. Palma-Tortosa S, Tornero D, Gronning Hansen M, Monni E, Hajy M, Kartsivadze S, et al. Activity in grafted human ips cell-derived cortical neurons integrated in stroke-injured rat brain regulates motor behavior. *Proc Natl Acad Sci U S A*. 2020;117:9094-9100
